# Symptomatic and Asymptomatic Transmission of SARS-CoV-2 in K-12 Schools, British Columbia, Canada April to June 2021

**DOI:** 10.1128/spectrum.00622-22

**Published:** 2022-07-06

**Authors:** Alexandra Choi, Louise C. Mâsse, Samantha Bardwell, Iryna Kayda, Yanjie Zhao, Yang Xin Zi Xu, Ani Markarian, Daniel Coombs, Adrienne Macdonald, Allison W. Watts, Nalin Dhillon, Michael Irvine, Collette O’Reilly, Pascal M. Lavoie, David M. Goldfarb

**Affiliations:** a Vancouver Coastal Health, Office of the Chief Medical Health Officer, Vancouver, British Columbia, Canada; b BC Children’s Hospital Research Institute, University of British Columbia, Vancouver, British Columbia, Canada; c School of Population and Public Health, University of British Columbia, Vancouver, British Columbia, Canada; d Department of Mathematics and Institute of Applied Mathematics, University of British Columbia, Vancouver, British Columbia, Canada; e Experimental Medicine Program, Faculty of Medicine, University of British Columbia, Vancouver, British Columbia, Canada; f Public Health Surveillance Unit, Vancouver Coastal Health, Vancouver, British Columbia, Canada; g Department of Pediatrics, Faculty of Medicine, University of British Columbia, Vancouver, British Columbia, Canada; h British Columbia Centre for Disease Control, Vancouver, British Columbia, Canada; i Vancouver School District, Vancouver, British Columbia, Canada; j BC Children’s and Women’s Health Centre, Vancouver, British Columbia, Canada; k Department of Pathology and Laboratory Medicine, University of British Columbia, Vancouver, British Columbia, Canada; Johns Hopkins Hospital

**Keywords:** COVID-19, SARS-CoV-2, school, transmission, asymptomatic

## Abstract

We prospectively studied SARS-CoV-2 transmission at schools in an era of variants of concern, offering all close contacts serial viral asymptomatic testing up to 14 days. From the 69 primary cases detected in schools, 392 close contacts were identified and offered asymptomatic testing. A total of 229 (58%) were close school contacts, and of these, 3 tested positive (1.3%), 2 of which were detected through asymptomatic testing. This is in contrast to the 117 household contacts, where 43 (37%) went on to become secondary cases. Routine asymptomatic testing of close contacts should be examined in the context of local testing rates, preventive measures, programmatic costs, and health impacts of asymptomatic transmission.

**IMPORTANCE** There is concern that schools may be a setting where asymptomatic infections might result in significant “silent” transmission of SARS-CoV-2, particularly after the emergence of more transmissible variants of concern. After the programmatic implementation of a strategy of asymptomatic testing of close COVID-19 contacts as part of contact tracing in the school setting, the majority of the secondary cases were still found to have occurred in home or social contacts. However, for the 6.2% of secondary cases that occurred in close school contacts, the majority were detected through asymptomatic testing. The potential added yield of this approach needs to be considered within the overall setting, including consideration of the local epidemiology, ongoing goals of case and contact management, additional costs, logistical challenges for families, and possible health impacts of asymptomatic transmission.

## INTRODUCTION

Fears of widespread, undetected asymptomatic transmission of COVID-19 in school settings have contributed to continued anxiety, large-scale testing, and restrictive measures in Europe and around the world. Consistent with contact tracing studies done internationally, Canada has observed limited transmission of SARS-CoV-2 among children in school settings (1, 2). However, infection in children typically results in mild illness, and many cases may be minimally symptomatic or asymptomatic (3). To assess asymptomatic transmission, a small number of studies have systematically tested contacts of students and staff members (4–6). While these studies have similarly detected minimal spread within schools, they have included a relatively low total number of index cases and were conducted before the emergence of more transmissible variants of concern (VOC). Moreover, these studies did not account for differential risk between classmates, tested full classes instead of prioritizing contacts with higher levels of exposure (e.g., deskmates/close friends), and could underestimate the real, material risk by inflating the denominator. The primary aim of this study was to assess the added yield of an asymptomatic serial testing program offered to all close contacts of primary cases, including those in the school setting.

## RESULTS

No schools were closed during the study period. Characteristics of primary cases and their close contacts are described in Table 1. During the study period, 69 primary cases were identified among K-12 students and staff. Of these, 23 (33%) were female, 46 (67%) were male, 65 (94%) were students, and 4 (6.2%) were staff. The majority of cases resided within Vancouver, British Columbia.

**TABLE 1:** Characteristics of 69 student and staff SARS-CoV-2 cases and all close contacts

Variable	Primary cases in students and staff (N = 69)	Close contacts who became cases^*b*^ (N = 48)	Close contacts who did not become cases (N = 344)
Age at time of report			
≤18	94% (65/69); median, 12; interquartile range (IQR), 9, 12; range, 6, 18	35% (17/48); median, 12; IQR, 7, 17; range, 4, 18	76% (262/344); median, 11; IQR, 9, 13; range, 1, 18
19–64	6% (4/69); median, 48; IQR, 41, 53; range, 35, 57	58% (28/48); median, 45; IQR, 38, 49; range, 21, 53	22% (77/344); median, 44; IQR, 37, 49; range, 19, 63
≥65	0	6% (3/48); median: 71; IQR, 71, 74; range, 71, 76	1% (5/344); median, 68; IQR, 67, 69; range, 67, 71
English first language	NA^*a*^	61% (25/41); 7 unknowns	81% (255/315); 29 unknowns
No. of bedrooms per home	NA	Median, 3; IQR, 3, 5; range, 1, 6; 9 unknowns	Median, 3; IQR, 3, 5; range, 1, 6; 273 unknowns
Linked to confirmed case or cluster			
No	50.72% (35/69)	4.17% (2/48)	NA
Yes, non-household contact	30.43% (21/69)	12.50% (6/48)	NA
Yes, household contact	18.84% (13/69)	83.33% (40/48)	NA
Case status			
Recovered/removed from isolation	66/69; 3 unknowns	48/48	NA
Deceased	0	0	NA
Active/lost to follow-up	0	0	NA
Ever hospitalized	0	4.17% (2/48)	NA
Ever admitted to ICU	0	0	NA
One or more comorbidities	1.45% (1/69)	14.58% (7/48)	NA

a NA, not applicable.

b Includes all contacts regardless of the setting in which they were exposed.

**FIG 1:**
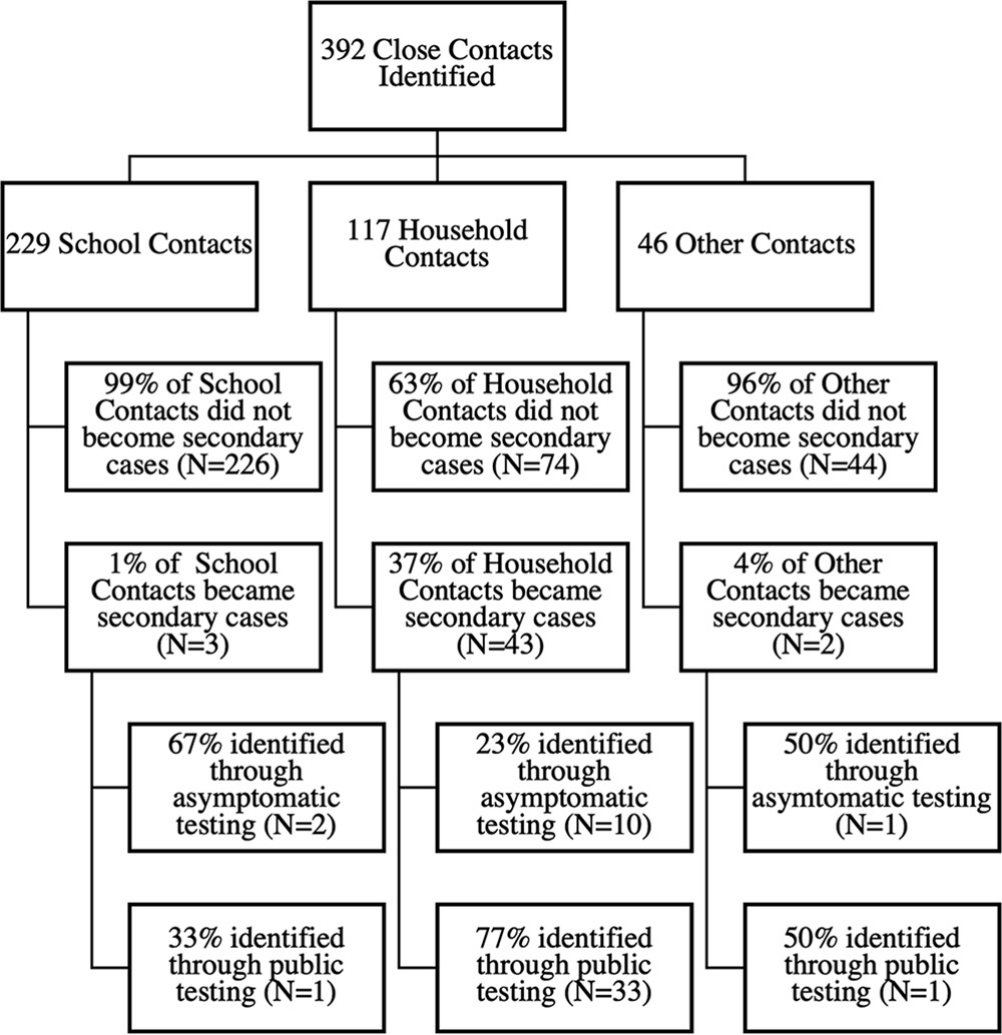
Flow diagram of close contacts who became cases and who did not by contact type. Other contacts comprise 48% social (N = 22), 7% extracurricular (N = 3), 35% mixed school and social (N = 16), and 10% unidentified (N = 5).

Based on the contact tracing of 69 primary cases, 392 close contacts were identified, offered asymptomatic testing, and instructed to self-isolate. Of these 392 close contacts, 229 were school contacts, 117 were household contacts, 22 were social contacts, 3 were extracurricular contacts, and 16 contacts were mixed (school and social), and the nature of the close contact could not be identified in 5 cases (Fig. 1). A total of 168 close contacts (43%) participated in asymptomatic testing, and 224 (57%) declined asymptomatic testing but agreed to participate in data collection. Of the 168 who agreed to asymptomatic testing, 13 (7.7%) tested positive. Of these 13 secondary cases, 2 were school contacts (15%), 10 were household contacts (77%), and 1 was a mixed school and social contact (8%). Of the 224 participants who declined asymptomatic testing, 35 (16%) of these later tested positive through the public symptomatic testing system. Of 229 school contacts, 66.3% spent over 1 h with the case, 83.6% interacted with the case indoors, and 56% wore a mask sometimes or not at all; 1.3% became secondary cases.

**FIG 2:**
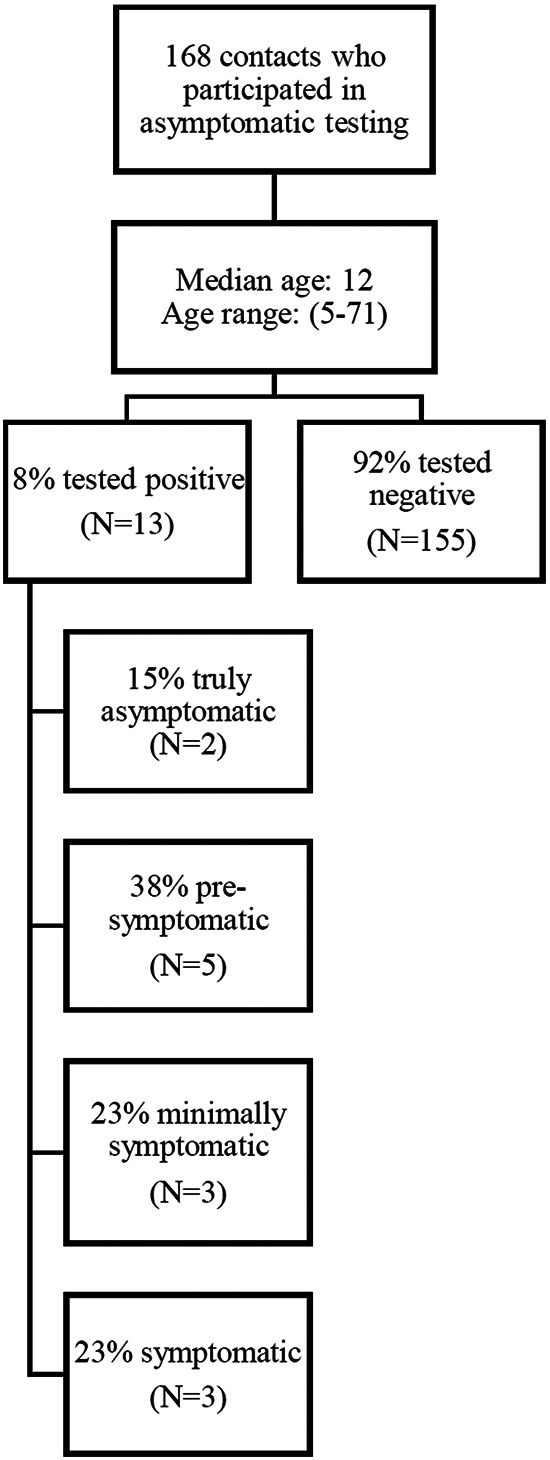
Flow chart of asymptomatic testing participation and test results.

Upon review of symptom data (Fig. 2), 5 of the 13 (38%) cases identified through asymptomatic testing were presymptomatic, meaning that they showed no symptoms at the time of close contact identification but developed symptoms later. Three (23%) were minimally symptomatic, describing discomfort or nonspecific illness but no discrete symptoms. A further 3 (23%) cases described clear symptoms. Two (15%) cases remained asymptomatic during the entire follow-up period (10 days after testing positive).

Aggregating the asymptomatic and symptomatic testing results in 392 close contacts, 48 (12%) were identified as secondary cases. The majority (43/48 cases, 90%) were household contacts. In follow-up of 229 school contacts with the closest interactions with the primary case, 3 (1.3%) became secondary cases.

## DISCUSSION

To our knowledge, our study is one of few that have systematically examined asymptomatic transmission in the context of VOC. We found a low proportion of secondary cases among close contacts of primary cases identified in the school setting, even accounting for asymptomatic cases. Moreover, we found that the majority of the transmission occurred outside of school and that household contacts had the highest secondary transmission rate. Of note, the secondary transmission rate in schools in our study was higher than that observed previously, which could be expected given that testing was limited to classmates with the closest interactions (7, 8). However, the yield of asymptomatic testing remained low even among classmates with prolonged close contact. Given that so few school contacts went on to become secondary cases, and the potential harms that can arise from missing school, these data question the value of isolation as a measure for controlling SARS-CoV-2 transmission within the school setting. The low numbers of social and extracurricular contacts identified reflected limitations placed on social gatherings and extracurricular activities during the study period and prevented risk comparisons.

Our results are generally in keeping with those of other studies that have sought to describe the epidemiology of SARS-CoV-2 in schools, with a recent meta-analysis of studies conducted prior to the Omicron era finding that child and youth index cases were 74% less likely to be associated with onward transmission than adult index cases (9). On the other hand, there is increasing evidence that the harms resulting from school closures are significant and may be felt well into the future (10).

A major strength of our study is that it includes data collection and prospective monitoring for a relatively large number of primary cases from both public and independent schools. However, our study is limited by the relatively low proportion (42.9%) of close contacts who agreed to undergo asymptomatic testing. A main limitation of the study is that it was conducted prior to the emergence of the omicron variant, which is known to have higher transmissibility; however, this study is one of the only contact tracing studies that has been conducted in the context of the emergence of VOC. Another limitation of the study is the short length of the study period. Low participation may reflect operational challenges that could be more pronounced in communities with existing barriers to health care. Further research investigating barriers to testing and subpopulations that may derive more benefit from enhanced testing may be warranted.

**Conclusion.** Results from enhanced and facilitated contact tracing and asymptomatic testing suggest low transmission of SARS-CoV-2 in K-12 schools with communicable disease prevention measures in the era of VOC. Two of three school contacts identified as secondary cases were found through asymptomatic testing; therefore, asymptomatic testing may be a useful adjunct to symptomatic testing, particularly where barriers to testing exist. Acknowledging resource requirements of asymptomatic testing, including sufficient staffing, availability of test kits, and test processing capacity, the benefits of asymptomatic testing may need to be balanced against barriers to participation and costs.

## MATERIALS AND METHODS

### Study setting including nonpharmaceutical interventions in schools.

Following closures in March 2020, K-12 schools in British Columbia reopened for the 2020/2021 academic year in September 2020. K-12 schools implemented COVID-19 safety plans developed with support from Public Health, which included public health measures, environmental measures, administrative measures, personal measures, and personal protective equipment (see supplemental material and details of prevention and control measures in published reports [1, 7]). Of note, during the period of study, nonmedical face masks were required (if tolerated) for all students from grade 4 and above as well as for all staff. Fig. S1 provides the COVID-19 daily rates per 100,000 population during the study period (12 April to 30 June 2021) in the health region where the schools were located. Despite the emergence of Alpha, Beta, and Gamma VOC, all schools remained open for the duration of the 2020/2021 school year. During the study period, there was codominance of Alpha and Gamma VOC in the Vancouver region.

### Identification of student and staff COVID-19 cases and their closest contacts.

Positive or indeterminate nucleic acid amplification tests (NAAT) and VOC screening results for Vancouver students and staff were automatically reported to Public Health and evenly distributed to two contact-tracing teams. This study included all lab-confirmed and lab-probable cases among students or staff and their contacts assigned to contact-tracing team two from 12 April 2021 until 30 June 2021. Case and contact definitions can be found in the supplemental material. Using a standardized form, contact tracers obtained informed consent and collected case and contact information through telephone interviews with students/staff members and guardians. This was supplemented by collateral information from school districts, principals, and teachers.

We longitudinally followed all close contacts, classroom contacts, and school contacts regardless of whether or not they underwent asymptomatic testing. For school exposures, individual risk assessments were conducted integrating cases’ symptoms, ages of cases and contacts, nature and duration of contact, mask use, setting (e.g., indoor/outdoor), and presence/absence of known SARS-CoV-2 transmission (8). Classmates specifically identified as close contacts by students, guardians, or staff were asked to undergo free, asymptomatic SARS-CoV-2 NAAT, self-isolate for 14 days, and self-monitor for symptoms. This typically included those who were close to the case during the school day, such as deskmates and close friends (11). Asymptomatic testing was done using serial, validated saline mouth rinse gargles at three time points: upon contact identification, 7 to 8 days after last exposure, and 10 to 14 days after last exposure (12). All other classroom contacts were asked to self-monitor for symptoms. If symptoms emerged, classroom and close contacts were directed to seek immediate, additional testing through public clinics that provided free, rapidly processed, widely accessible symptomatic testing. Entire classes were isolated and offered testing in the event of a school cluster where transmission may have occurred within the classroom.

To characterize clusters, primary cases were defined as laboratory-confirmed or laboratory-probable cases with the earliest symptom onset dates within the school setting (supplemental material). Where a classmate of a primary case tested positive, linkage was assumed. Where contacts of classmates tested positive (e.g., family members of a classmate), repeat asymptomatic testing of the classmate was requested and exposure through the primary student/staff case was assumed.

### Ethical statement.

This project was reviewed by the BC Children’s and Women’s Research Ethics Board and deemed a QA/QI activity; it was therefore exempted from formal ethical review but received a privacy impact assessment (no. PIA 2021-40). Informed consent was obtained from all participants or their guardians.
